# A role for the circadian photoreceptor CRYPTOCHROME in regulating triglyceride metabolism in *Drosophila*

**DOI:** 10.1093/g3journal/jkae220

**Published:** 2024-09-12

**Authors:** Swetha Gopalakrishnan, Sanjay Ramnarayan Yadav, Nisha N Kannan

**Affiliations:** Chronobiology Laboratory, School of Biology, Indian Institute of Science Education and Research, Thiruvananthapuram, Kerala 695551, India; Chronobiology Laboratory, School of Biology, Indian Institute of Science Education and Research, Thiruvananthapuram, Kerala 695551, India; Chronobiology Laboratory, School of Biology, Indian Institute of Science Education and Research, Thiruvananthapuram, Kerala 695551, India

**Keywords:** cryptochrome, *Drosophila*, metabolism, triglyceride, feeding, starvation, lifespan

## Abstract

The biological rhythms generated by the endogenous circadian clocks across the tree of life regulate numerous behavioral, metabolic, and physiological processes. Although evidence from various studies in *Drosophila melanogaster* indicates the importance of the core circadian clock genes in the intricate interplay between the circadian clock and metabolism, little is known about the contribution of the circadian photoreceptor/s in this process. The deep brain circadian photoreceptor CRYPTOCHROME (CRY) is essential for resetting the clock in response to light and is also highly expressed in metabolically active tissues in *Drosophila*. In this study, we sought to explore the possible roles played by CRY in triglyceride (TG) metabolism. We observed that the *cry* mutant (*cry^01^*) flies exhibited increased starvation resistance and TG levels under both 12-hour (h) light:12-h dark cycle (LD) and under constant light compared with the control *w^1118^* flies. We also observed that *cry^01^* flies had significantly increased food intake, glycogen concentrations, and lifespan under LD. In addition, *cryptochrome* seemed to affect TG levels in adult flies in response to calorie-restricted and high-fat diets. These results suggest a role for the circadian photoreceptor CRY in TG metabolism in *Drosophila*.

## Graphical Abstract

**Graphical Abstract:**
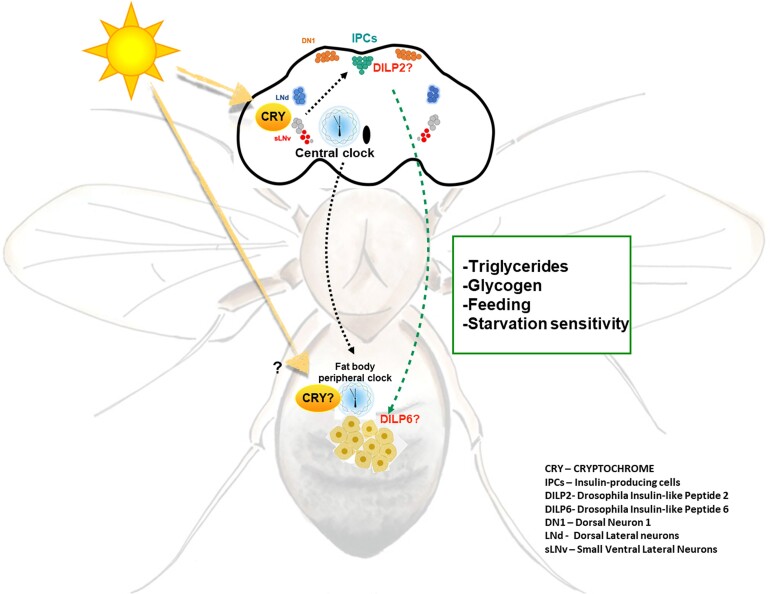
Example of the SCORE2 Asia-Pacific model recalibrated to all four risk regions in the Asia-Pacific region, highlighting regional differences in cardiovascular disease (CVD) risk profiles. HDL-c, high-density lipoprotein cholesterol; SBP, systolic blood pressure; WHO GHE, World Health Organization’s Global Health Estimates.

## Introduction

Circadian rhythms are the nearly 24-hour (h) innate, endogenous rhythms that are fairly conserved and give rise to rhythms in physiological processes in most organisms ranging from photosynthetic and nonphotosynthetic bacteria to humans (Rosbash 2009; Eelderink-Chen *et al*. 2021). These rhythms arise from the molecular clocks that allow organisms to anticipate and prepare for environmental changes and synchronize their behavior, physiology, and metabolism according to the external environment (Sharma and Chandrashekaran 2005). External cues such as light, temperature, and timing of food entrain the circadian clock and are termed “zeitgebers” (Daan and Aschoff 2001).

Pioneering studies by Konopka and Benzer (1971) identified the first circadian clock gene *period* (*per*) in *Drosophila.* The circadian transcription factors CLOCK (CLK) and CYCLE (CYC) form a heterodimeric complex and promote the transcription of the core clock genes *per* and *timeless* (*tim*) and make up the positive limb of the transcriptional–translational feedback loop (TTFL). PER and TIM accumulate during the night and form a heterodimer, enter the nucleus, and inhibit the activity of CLK/CYC (Menet *et al.* 2010). Thus, the core clock proteins PER and TIM act as the transcriptional repressors and make up the negative limb of the TTFL in the circadian clock. The molecular clock, influenced significantly by environmental cues, particularly light, is entrained through the photoreceptor CRYPTOCHROME (CRY). CRY undergoes a conformational change upon photon absorption, enabling it to bind to TIM (Ozturk *et al.* 2011). This interaction facilitates TIM ubiquitination and proteasomal degradation, effectively resetting the clock, and subsequently, PER is degraded as well (Peschel *et al.* 2009; Tataroglu and Emery 2014; Lin *et al.* 2023). Loss-of-function *cry* mutant (*cry^01^*) flies exhibit behavioral split rhythms with long and short free-running components under constant light (LL), whereas a genetically normal fly typically goes arrhythmic under LL (Dolezelova *et al*. 2007).

The central circadian clock in *Drosophila* comprises about 150 clock neurons in the brain (Reinhard *et al.* 2022). In addition to the central circadian clock in the *Drosophila* brain, there are peripheral clocks in several tissues that keep time (Allada and Chung 2010). Some of these peripheral clocks regulate rhythms in a tissue-specific manner coordinating with the central pacemaker. Multiple studies in *Drosophila* and mammals have shown that the loss of synchrony between these central and peripheral clocks is associated with numerous deleterious consequences on metabolism (Schäbler *et al.* 2020; Marcheva *et al.* 2010; Turek *et al.* 2005; Rudic *et al.* 2004).

Some studies also indicate a role for the central clock neurons in controlling energy homeostasis, especially lipid storage in *Drosophila* (DiAngelo et al. 2011). The central clock neurons may communicate with the fat body peripheral clock to regulate lipid storage and feeding behavior (Xu *et al*. 2008). Fat body clock drives rhythmic expression of genes involved in metabolism (Xu *et al.* 2011). *Drosophila* insulin-like peptides (DILPs) secreted by the insulin-producing cells (IPCs) in the pars intercerebralis (PI) region drive the rhythmic expression of metabolic transcripts in the fat body and are required to drive the feeding rhythm. Signaling from central clock neurons to IPCs is also relevant for this feeding rhythm and food intake (Barber *et al.* 2016).

The studies that have explored the bidirectional interaction between circadian clocks and metabolism in *Drosophila* have primarily focused on the core clock components, and there is not much knowledge about the role played by the circadian photoreceptor/s in this process (Sehgal 2016). The blue light circadian photoreceptor CRY in *Drosophila* has been conventionally shown to facilitate the light-mediated entrainment and resetting of the central circadian clock in the brain (Emery *et al.* 1998). CRY is also required for the normal oscillation of peripheral clocks in certain tissues such as the antennae and malpighian tubules (Krishnan *et al.* 2001; Ivanchenko *et al.* 2001). Collins *et al.* 2006 suggested that CRY can act as a transcriptional repressor in the eyes when both PER and CRY were overexpressed. Additionally, peripheral circadian clocks in *Drosophila* are able to perceive light (Plautz *et al.* 1997;Ito and Tomioka 2016). Despite *cry* being expressed in the metabolically active tissues such as the gut, fat body (Leader *et al.* 2018) and showing rhythmic expression in the fat body (Xu *et al.* 2011), there haven’t been a lot of studies that have attempted to explore the role of CRY in metabolism. A previous study revealed a role for circadian photoreceptor CRY in regulating feeding in *Drosophila* (Seay and Thummel 2011). The results of our present study show that CRY does play a role in governing triglyceride (TG) metabolism and starvation resistance in *Drosophila*.

## Methods

### Fly strains and maintenance

The following fly lines were used in this experiment, *w^1118^* (BDSC #5905) and *cry^01^* (Dolezelova *et al.* 2007). All the fly stocks were maintained on standard cornmeal dextrose medium in an incubator (MIR-154, Panasonic) at 25°C temperature, ∼450 lux light intensity, 70 ± 5% humidity, and 12-h light:12-h dark cycle (LD) or in LL wherever applicable. The lights in the incubator came up at zeitgeber time 00 (ZT00) and went off at ZT12 under LD. Fifty first-instar larvae were collected in fresh food vials avoiding overcrowding within 2–3 h of hatching. Freshly emerged male flies were collected from this (15–16 per vial) and used for the experiments. *cry^01^* flies backcrossed into the *w^1118^* background for 5 generations were used for most of the experiments. To test whether the role of *cry* in TG metabolism is clock-dependent, we used *per^01^*, *cry^01^*, and *per^01^;;cry^01^* flies backcrossed into the *Canton-S* background (obtained from Dr. Charlotte Förster's Lab, University of Wurzburg). We used FlyBase (release FB2024_04) to find information on the phenotypes and gene expression (Öztürk-Çolak *et al.* 2024).

### PCR genotyping

To confirm the *per^01^* mutation in the *per^01^* and *per^01^;;cry^01^* flies, genomic DNA was extracted from the flash-frozen flies using Invitrogen Jetflex Genomic DNA purification kit using the manufacturer's protocol. A segment of the *per* gene was then PCR-amplified using the master mix from Takara Bio. The PCR was carried out in 20 μl of reaction mixture containing 10 μl of master mix, 1 μl of 10 μM forward and reverse primers, respectively (primer sequence mentioned in Supplementary Table 1), and 100–200 ng of template DNA. The final volume was made up with nuclease-free water. The amplification reaction was performed in a thermal cycler (SimpliAmp, Thermo Fisher) using the PCR conditions with the steps of initial denaturation at 95°C for 3 minutes (min) followed by 35 cycles of 95°C for 30 seconds (sec), annealing at 58°C for 15 sec, and 72°C for 30 sec and a final extension at 72°C for 10 min. The amplified PCR product was subjected to partial restriction digestion using XbaI enzyme (Fast Digest ThermoScientific, digestion site: T^CTAGA) since the mutation in the *per* gene introduced a restriction site for XbaI. The reaction was incubated at 37°C for 30 min followed by enzyme inactivation at 65°C for 20 min (Gamal 2022).

### Activity-rest rhythm recording

Two to three-day-old male flies were transferred into locomotor activity glass tubes containing cornmeal dextrose medium, and the activity-rest rhythms were recorded by using the *Drosophila* Activity Monitors (Trikinetics, USA) for the first 5 days under LD in a cooled incubator (MIR-154, Panasonic, Japan). On day 6, these flies were flipped into another set of locomotor activity glass tubes containing fresh cornmeal dextrose medium just before ZT00 and the activity-rest rhythms were recorded for 10 days under LL. 32 flies per genotype were used for the recordings. The data obtained were analyzed using the software ClockLab (Actimetrics, USA) to visualize the actograms.

### Media composition for calorie restriction and high-fat diet

#### Calorie restriction

*Drosophila* normal diet (ND) control media contained cornmeal (5.82%), dextrose (5.08%), inactive yeast (2.36%), agar (0.8%), and nipagin (10% w/v in ethanol). Calorie-restricted diet (CRD) media contained cornmeal (2.91%), dextrose (2.54%), and inactive yeast (1.18%), while agar and nipagin were the same as the control. Freshly emerged male flies were fed these media for 5 days and then used for the experiments performed under LD.

#### High-fat diet

*Drosophila* ND control media contained cornmeal (5.82%), dextrose (5.08%), inactive yeast (2.36%), agar (0.8%), and nipagin (10% w/v in ethanol). High-fat diet (HFD) media contained the same composition of all the components, along with 10% virgin coconut oil mixed with an additional 7.5% agar. Freshly emerged male flies were fed these media for 5 days and then used for the experiments performed under LD.

### Starvation sensitivity assay

Five-day-old male flies were transferred to vials containing 1% agar, and the number of dead flies was counted every 2 h under LD and LL. 3–4 biological replicates containing 4–6 technical replicates were taken for each experiment. One technical replicate refers to a vial with ∼16 flies.

### TG assay

Five-day-old male flies were sampled at ZT14 and homogenized in 0.05% Tween-20 (cat. no. P2287, Sigma) using Bullet Blender Storm (BBY24M from Next Advance). The homogenate was heat-inactivated at 70°C for 5 min and centrifuged at 14,000 rpm for 3 min (Teleman *et al.* 2005a). Sigma triglyceride kit (act. no. TR0100) from Sigma-Aldrich was used to assess TG level, and Quick Start Bradford 1× Dye Reagent (cat. no. 500-0205) from Bio-Rad was used for protein estimation. This was followed by colorimetric estimation using TECAN Infinite M200 pro-multimode plate reader in 96-well format. The absorption maximum of 540 and 595 nm was used for TG and protein content, respectively, and the TG levels were quantified as the % ratio of TG to total protein levels (Pathak and Varghese 2021). For checking the TG utilization, flies were transferred to vials containing 1% agar, were sampled at 00, 12, 15, 18, and 24 h poststarvation, and the TG levels were assayed. 11–15 biological replicates, each containing 5 flies, were used for the TG measurements.

### Glycogen assay

Sample preparation for glycogen measurement was similar to TGs, following the manufacturer's protocol (cat. no. MAK016 from Sigma-Aldrich). The absorbance was measured at 570 nm using a TECAN Infinite M200 pro-multimode plate reader. 11–15 biological replicates, each containing 5 flies, were used for the experiments.

### Feeding assay

Five-day-old flies were fed for 30 min at ZT01 and ZT13 under LD and at Circadian Time (CT) 01 and CT13 under LL with yeast paste containing Orange G dye (cat. no. 1936-15-8) from Sigma-Aldrich. The flies were starved by transferring to vials containing 1% agar 12 h prior to the assay. After feeding, the flies were flash-frozen (5 flies per tube) and homogenized using 0.05% Tween-20. The homogenate was analyzed colorimetrically at 478 nm using TECAN Infinite M200 pro-multimode plate reader in the 96-well format. The absorbance of the homogenate was directly proportional to the food intake. A detailed protocol is given in Sudhakar *et al.* (2020). The feeding experiments were replicated independently, and data from 3 such biological replicates were analyzed.

### Lifespan assay

Freshly emerged 10 male flies were transferred into vials containing cornmeal dextrose ND medium. Three biological replicates, each consisting of 10–12 such replicate vials, were set up under LD in a cooled incubator (Percival Scientific, Perry, IA, USA). Each replicate vial contained 10 flies. Flies were provided with fresh food medium every third day to avoid death due to desiccation. The death that occurred on each day was noted until all flies died, and survival curves were analyzed using the log-rank test (Han *et al*. 2016).

### Quantitative RT-PCR

Five-day-old males entrained to LD were used for mRNA isolation. Three biological replicates each with 30 flies were sampled at ZT02 for *cry, brummer* (*bmm*)*, 4ebp, dilp2,* and *dilp6* and at ZT14 only for *per*. 30 fly heads or 10 fly bodies were used for the RNA isolation by the TRIzol method (Toni *et al.* 2018). Detailed protocol is given in Anna *et al.* (2023). The housekeeping gene *rp49* was used as the reference gene, and the mRNA values shown are relative to *rp49* mRNA. The primer sequences used are listed in Supplementary Table 1.

### Statistical analyses

Student's unpaired *t*-test/Welch's *t*-test, one-way ANOVA/Brown–Forsythe and Welch ANOVA, and two-way ANOVA followed by post hoc Tukey's HSD/Dunnett's multiple comparisons were used when data were normally distributed. Kruskal–Wallis test followed by Dunn's post hoc multiple comparisons or Mann–Whitney test was used for datasets that did not have a normal distribution. Log-rank test (Han *et al*. 2016) was used for analyzing survival curves. To calculate the Δtriglyceride/protein (%), empirically obtained data from 3 biological replicates were subjected to bootstrapping, where the data were re-sampled with replacement to generate 4 replicate sets of data (Good 2012). The statistical analyses were performed using GraphPad PRISM version 10.2.3. The error bars in all the graphs represent the SEM. In all the figures, * represents *P* < 0.05, **P < 0.01, ***P < 0.001, and ****P < 0.0001.

## Results

### Circadian photoreceptor CRYPTOCHROME regulates starvation sensitivity and TG levels in *Drosophila*

In order to understand the role of *cryptochrome* in metabolism in *Drosophila*, we first went about checking the sensitivity to starvation of loss-of-function *cry* mutant. The starvation sensitivity assay serves as a preliminary assay to get to know the changes in the metabolic landscape of the fly. To this end, we backcrossed *cry^01^* flies into the *w^1118^* background for 5 generations and confirmed that the backcrossed *cry^01^* flies possess significantly reduced *cry* transcript levels compared with the *w^1118^* controls (Supplementary Fig. 1a). We recorded the activity-rest rhythms of these flies under LD for 5 days followed by LL for 10 days and confirmed that *cry^01^* flies show split rhythmicity under LL as described in Dolezelova *et al.* (2007), whereas the *w^1118^* control flies were arrhythmic (Supplementary Fig. 1b, c).

**Fig. 1.**
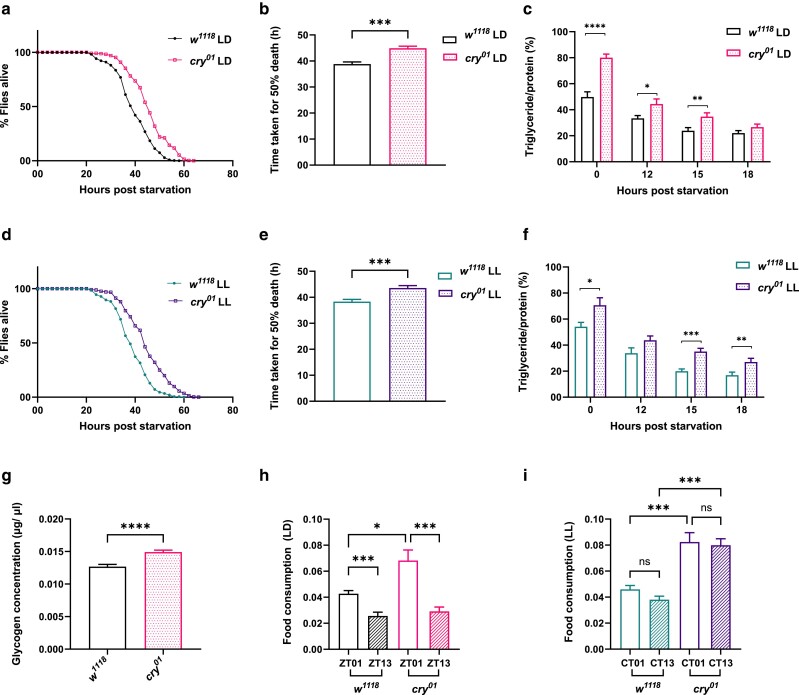
Effect of light and cryptochrome on starvation sensitivity, TG utilization, glycogen levels, and feeding. a) Survival curve for *w^1118^* and *cry^01^* flies under LD, data shown as the percentage of flies which were alive at various time points of starvation. b) Time taken for 50% fly death under starvation for *w^1118^* and *cry*^*01*^ flies under LD. c) Utilization of TGs at different stages of starvation in *w^1118^* and *cry^01^* flies under LD. d) Survival curve for *w^1118^* and *cry^01^* flies under LL, data shown as the percentage of flies which were alive at various time points of starvation. e) Time taken for 50% fly death under starvation for *w^1118^* and *cry^01^* flies under LL. f) Utilization of TGs at different stages of starvation in *w^1118^* and *cry^01^* flies under LL. g) Glycogen concentration (μg/μl) in ad libitum-fed *w^1118^* and *cry^01^* flies under LD. h) Food consumption under LD in response to 12 h of starvation for *w^1118^* and *cry^01^* flies at ZT01 and ZT13. i) Food consumption under LL in response to 12 h of starvation for *w^1118^* and *cry^01^* flies at CT01 and CT13.

Subsequently, we performed a starvation sensitivity assay in 5-day-old *w^1118^* and *cry^01^* flies under LD. The results showed that the *cry^01^* flies survived significantly longer under starvation than the *w^1118^* flies (Fig. 1a, *P* < 0.0001, log-rank test). There was also a significant difference in the time taken for 50% fly death (Fig. 1b, *P* < 0.001, Mann–Whitney test). Next, we went on to measure the TG utilization under LD at 00 (ZT14), 12, 15, and 18 h poststarvation and saw that the *cry^01^* flies had significantly higher TG levels than *w^1118^* flies at 00, 12, and 15 h poststarvation (Fig. 1c, *P* < 0.0001 for 00 h *w^1118^* vs *cry^01^*, *P* = 0.025 for 12 h *w^1118^* vs *cry^01^*, *P* = 0.008 for 15 h *w^1118^* vs *cry^01^*, *P* = 0.147 for 18 h *w^1118^* vs *cry^01^* by Student's *t*-test). We also measured the weights of the flies and did not find any significant difference in the weights (Supplementary Fig. 1d).

Since *cry* in *Drosophila* has a conventional light-sensing role, we wanted to see the effects of loss of function of *cry* on TG metabolism under LL. Under LL, *w^1118^* flies typically exhibit arrhythmicity in activity-rest rhythm and *cry^01^* flies exhibit split rhythmicity. Similar to what we observed under LD, we saw that the *cry^01^* flies fared better when starved compared with the control (Fig. 1d, *P* < 0.0001 by log-rank test) and *cry^01^* flies had a significant increase in time taken for 50% fly death under starvation (Fig. 1e, *P* < 0.001, Mann–Whitney test). When the TG utilization was quantified, the *cry^01^* flies had significantly more TG levels than controls at 00, 15, and 18 h poststarvation (Fig. 1f, *P* = 0.023 for 00 h *w^1118^* vs *cry^01^*, *P* = 0.052 for 12 h *w^1118^* vs *cry^01^*, *P* < 0.0001 for 15 h *w^1118^* vs *cry^01^*, *P* = 0.011 for18 h *w^1118^* vs *cry^01^* by Student's *t*-test). These results suggest that *cry* regulates the levels of TG and starvation sensitivity in *Drosophila*.

### CRYPTOCHROME affects glycogen levels and feeding in flies

Following starvation sensitivity and TG assays, we were further interested in understanding whether CRY affects other stored energy reserves such as glycogen and also the feeding rhythm of the flies. When glycogen concentrations were measured under LD, *cry* mutants seemed to have increased glycogen levels compared with the control flies (Fig. 1g, *P* < 0.0001 by Mann–Whitney test). We assessed the food intake in 5-day-old *w^1118^* and *cry^01^* flies under LD at ZT 01 and at ZT13. Previous studies showed diurnal rhythmicity in feeding behavior with a peak in the early daytime in *Drosophila* (Xu *et al.* 2008). Under LD, we observed that at ZT01, *cry^01^* flies fed significantly more than *w^1118^* flies. At ZT13, we observed that both *w^1118^* and *cry^01^* flies fed significantly less compared with the *w^1118^* and *cry^01^* flies at ZT01, respectively (Fig. 1h, one-way ANOVA test was performed and the *P*-values are as follows: *P* < 0.001 for *w^1118^* ZT01 vs *w^1118^* ZT13, *P* < 0.001 for *cry^01^* ZT01 vs *cry^01^* ZT13, *P* = 0.03 for *w^1118^* ZT01 vs *cry^01^* ZT01). Under LL, we assessed the food intake at CT01 and CT13; there was not a significant difference in the food consumption at CT01 and CT13 in both *w^1118^* and *cry^01^* flies, indicating abolished feeding rhythms under LL at least in *w^1118^* flies. Further, *cry^01^* flies seem to feed significantly more at both CT01 and CT13 compared with *w^1118^* flies under LL (Fig. 1i, *P* < 0.001 for *w^1118^* CT01 vs *cry^01^* CT01, *P* < 0.001 for *w^1118^* CT13 vs *cry*^01^ CT13, *P* > 0.99 for *w^1118^* CT01 vs *w^1118^* CT13, *P* > 0.99 for *cry^01^* CT01 vs *cry^01^* CT13 by Kruskal–Wallis test). These results suggest that CRY affects the food intake at ZT01 and consequently the feeding rhythm as well as affects the stored glycogen levels in *Drosophila* under LD.

### *Dilp6* and *4ebp* transcript levels are reduced in *cryptochrome* mutants

**Fig. 2.**
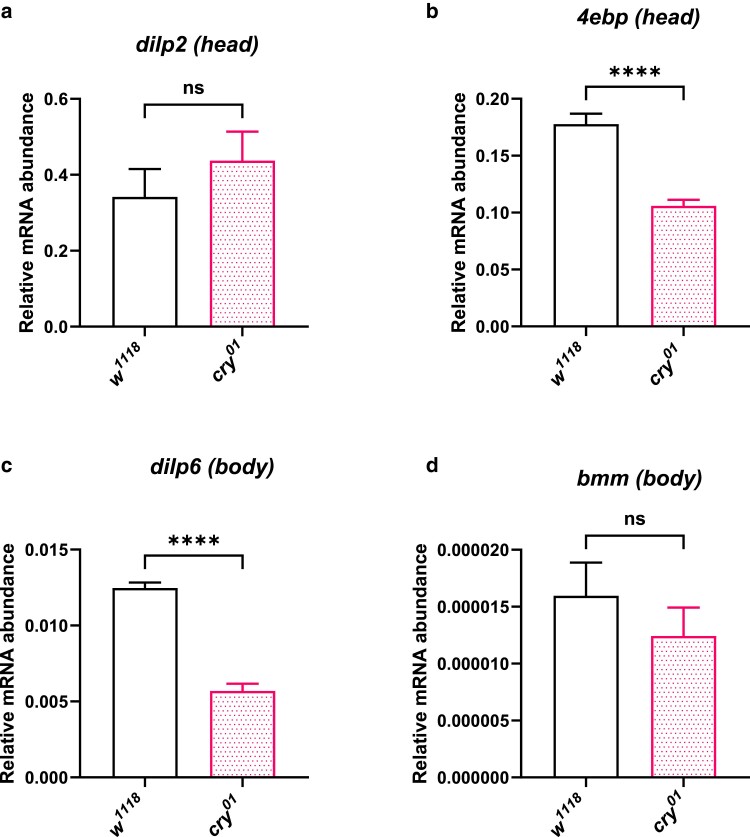
Cryptochrome affects transcript levels of *4ebp* and *dilp6.* a) Relative mRNA abundance of *dilp2* in *w^1118^* and *cry^01^* heads at ZT02. b) Relative mRNA abundance of *4ebp* in *w^1118^* and *cry^01^* heads at ZT02. c) Relative mRNA abundance of *dilp6* in *w^1118^* and *cry^01^* bodies at ZT02. d) Relative mRNA abundance in *bmm* in *w^1118^* and *cry^01^* bodies at ZT02.

Central circadian clocks regulate feeding rhythms and energy homeostasis in *Drosophila* by signaling to other peripheral tissues (He *et al.* 2023). Barber *et al.* (2016) showed that insulin mediates circadian output through the signaling between the central circadian clock dorsal clock neurons (DN1) and the IPCs in the PI. The activity and firing rates of IPCs are in turn regulated by feeding. When we checked the transcript levels of insulin-like peptide 2 (*dilp2*), there was no statistically significant difference in the *dilp2* transcript levels between the *w^1118^* flies and *cry^01^* fly heads at ZT02 (Fig. 2a). It has been previously shown that heightened insulin signaling promotes lipid accumulation in *Drosophila* through inhibition of the transcription factor Forkhead box O (FOXO) (DiAngelo and Birnbaum 2009). Since we observed an increase in TG levels in *cry^01^* flies, we decided to check the transcript levels of some of the FOXO targets that regulate fat metabolism—*Drosophila* insulin-like peptide-6 (*dilp6*) expressed in the fat body, *4ebp* (eukaryotic translation initiation factor 4E-binding protein), and the lipase *brummer* (*bmm*) (Bai *et al.* 2012; Teleman *et al*. 2005b; Grönke *et al.* 2005) in *cry^01^* flies. We observed that *dilp6* transcript levels in the body and *4ebp* transcript levels in the head were significantly reduced in *cry^01^* flies at ZT02 (Fig. 2b, c, *P* < 0.0001 by unpaired Student's *t*-test). The transcript levels of the lipase *bmm* in the body did not change significantly between *cry^01^* and *w^1118^* flies at ZT02 (Fig. 2d). A reduction in the transcript levels of FOXO targets *dilp6* and *4ebp* might indicate an elevated insulin signaling in the *cry^01^* flies which needs to be further confirmed by estimating the DILP2 levels in the hemolymph.

### Role of the circadian clock in mediating the effects of CRY on TG metabolism

**Fig. 3.**
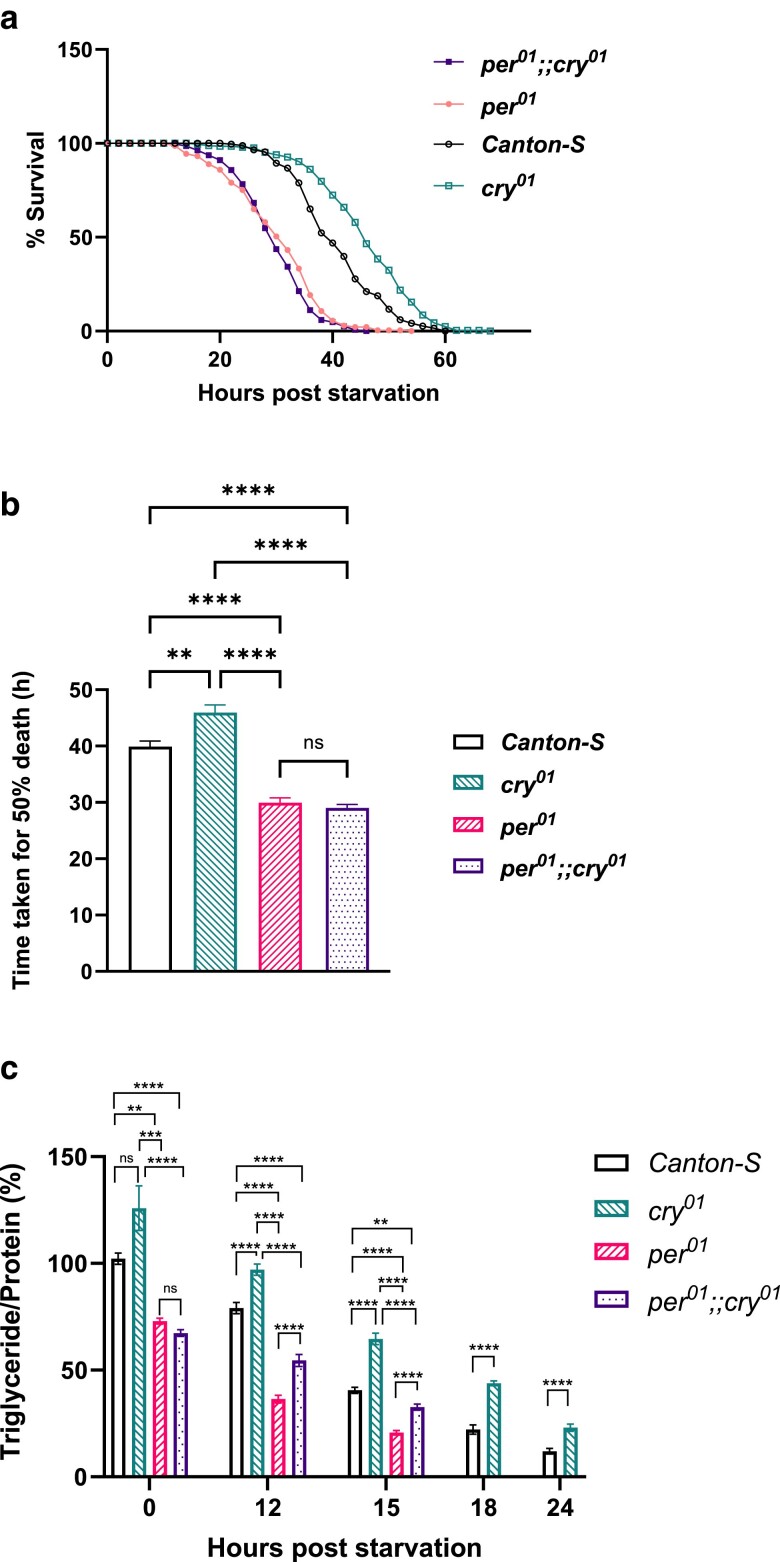
Role of the circadian clock in mediating the effects of CRY on TG metabolism. a) Survival curve for *Canton-S, per^01^, per^01^;;cry^01^, and cry^01^* flies under LD, data shown as the percentage of flies which were alive at various time points of starvation. b) Time taken for 50% fly death under starvation for *Canton-S, cry^01^, per^01^,* and *per^01^;;cry^01^* under LD. c) TG utilization in *Canton-S, cry^01^, per^01^,* and *per^01^;;cry^01^* flies under LD at 00, 12, 15, 18, and 24 h poststarvation (*per^01^* and *per^01^;;cry^01^* flies were not used for assaying TG utilization at 18 and 24 h poststarvation since they start dying around 18 h).

In order to understand whether the effects exerted by CRY on the TG metabolism in *Drosophila* are dependent or independent of the circadian clock, starvation sensitivity and TG levels were assayed in *per^01^*, *cry^01^* single mutants and in the *per^01^;;cry^01^* double-mutant flies with *Canton-S* background control. To confirm the genotypes of the mutants, the transcript levels of *cry* were checked in the *cry^01^* and the *per^01^;;cry^01^* double mutants and PCR genotyping was done to confirm the mutation in the *per* gene in the *per^01^* and in the *per^01^;;cry^01^* double mutants. The *cry* transcript level was significantly reduced in the *cry^01^* and the double-mutant *per^01^;;cry^01^* flies compared with *Canton-S* at ZT02 (Supplementary Fig. 1e, *P* < 0.0001 for *Canton-S* vs *cry^01,^ P* < 0.0001 for *Canton-S* vs *per^01^;;cry^01^* by Brown–Forsythe and Welch ANOVA). In *per^01^* flies, a point mutation on the exon 4 of the *per* gene introduces a premature stop codon. The presence of *per^01^* mutation was confirmed by PCR genotyping where PCR was performed followed by restriction digestion. *per^01^* and *per^01^;;cry^01^* flies had the predicted digestion fragments (∼178 and 282 bp), while there was a single undigested PCR fragment of around 460 bp in the *Canton-S* control (Supplementary Fig. 1f, g). The details are provided in the *Methods* section). When subjected to starvation, *cry^01^* flies seemed resistant to starvation compared with *Canton-S, per^01^*, and the *per^01^;;cry^01^* flies (Fig. 3a, *P* < 0.001 for *Canton-S* vs *cry^01^*, for *cry^01^* vs *per^01^*, and for *cry^01^* vs *per^01^;;cry^01^* by log-rank test). The time taken for 50% fly death was significantly more for *cry^01^* flies compared with *Canton-S, per^01^*, and the *per^01^;;cry^01^* flies (Fig. 3b, *P* = 0.0081 for *Canton-S* vs *cry^01^* and *P* < 0.0001 for *cry^01^* vs *per^01^* and for *cry^01^* vs *per^01^;;cry^01^* by Brown–Forsythe and Welch ANOVA). Both *per^01^* and *per^01^;;cry^01^* flies were sensitive to starvation compared with the *Canton-S* and *cry^01^* flies (Fig. 3b, *P* < 0.0001 for *Canton-S* vs *per^01^*, for *Canton-S* vs *per^01^;;cry^01^*, for *cry^01^* vs *per^01^*, and for *cry^01^* vs *per^01^;;cry^01^* by Brown–Forsythe and Welch ANOVA), and there was not a significant difference in starvation sensitivity and the time taken for 50% fly death between *per^01^* and *per^01^;;cry^01^* flies (Fig. 3b, *P* = 0.9456 by Brown–Forsythe and Welch ANOVA). TG utilization was assayed at 00, 12, 15, 18, and 24 h poststarvation for *Canton-S* and *cry^01^* flies and at 00, 12, 15, and 18 h poststarvation for *per^01^* and *per^01^;;cry^01^* flies since these flies were quite sensitive to starvation and started dying around 18 h poststarvation. *cry^01^* flies had significantly increased TG levels at 12, 15, 18, and 24 h poststarvation compared with the *Canton-S* flies (*P* < 0.0001 for *Canton-S* vs *cry^01^* at 12, 15, 18, and 24 h. The details of the statistical tests are given in Supplementary Table 2). Although *cry^01^* flies had increased TG levels at the start of starvation (00 h) when fed ad libitum, this difference did not reach a statistically significant level when compared with the control *Canton-S* flies (*P* > 0.9999 for *Canton-S* vs *cry^01^*). In contrast, *per^01^* flies had significantly decreased TG levels compared with both *Canton-S* and *cry^01^* flies at 00, 12, and 15 h poststarvation (*P* = 0.001 for *Canton-S* vs *per^01^* at 00 h, *P* < 0.0001 for *Canton-S* vs *per^01^* at 12 and 15 h, *P* < 0.0001 for *cry^01^* vs *per^01^* at 00, 12, and 15 h). *per^01^;;cry^01^* double-mutant flies also had significantly decreased TG levels compared with both *Canton-S* and *cry^01^* flies at 00, 12, and 15 h poststarvation (*P* < 0.0001 for *Canton-S* vs *per^01^;;cry^01^* at 00 and 12 h and *P* = 0.0049 for *Canton-S* vs *per^01^;;cry^01^* at 15 h, *P* < 0.0001 for *Canton-S* vs *per^01^* at 12 and 15 h, *P* < 0.0001 for *cry^01^* vs *per^01^;;cry^01^* at 00, 12, and 15 h). There was not a significant difference in the TG levels between *per^01^* and *per^01^;;cry^01^* flies when fed ad libitum (*P* > 0.9999). However, there was a difference in the TG utilization during the course of starvation between *per^01^* and *per^01^;;cry^01^* flies, and we observed that *per^01^;;cry^01^* flies had significantly more TG levels compared with *per^01^* flies at 12 and 15 h poststarvation (*P* < 0.0001 for *per^01^;;cry^01^* vs *per^01^* at 12 and 15 h). Taken together, these results imply that a functional circadian clock is likely required for the metabolic effects seen in *cry^01^* flies previously.

### CRYPTOCHROME affects lifespan in ad libitum-fed flies

**Fig. 4.**
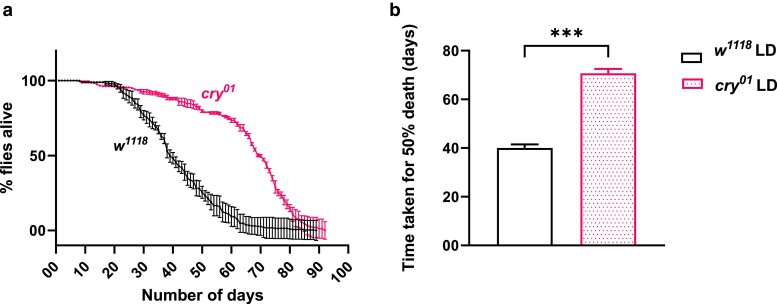
Effect of *cryptochrome* mutation on lifespan in flies. a) The lifespan of *w^1118^* and *cry^01^* flies with the percentage of flies alive plotted on the *y*-axis and time in days plotted on the *x*-axis. b) Time taken for 50% fly death (in days) for ad libitum-fed *w^1118^* and *cry^01^* flies under LD.

Since we observed that the *cry* mutants had significantly increased resistance to starvation and TG levels, we wanted to check whether the absence of *cry* also confers a survival benefit for the flies and went on to assay the lifespan. We found that the *cry^01^* flies lived significantly longer than the *w^1118^* flies under ad libitum-fed conditions under LD (Fig. 4a, *P* < 0.0001 by log-rank test). On quantifying the time taken for 50% death, we found that it was 40.03 ± 1.44 (mean ± SEM) days for *w^1118^* flies and 70.66 ± 1.82 (mean ± SEM) days for *cry^01^* flies (Fig. 4b, *P* < 0.001, Mann–Whitney test). These results suggest that *cry* mutation increases the lifespan in *Drosophila*.

### CRYPTOCHROME affects TG levels in *Drosophila* in response to calorie restriction

**Fig. 5.**
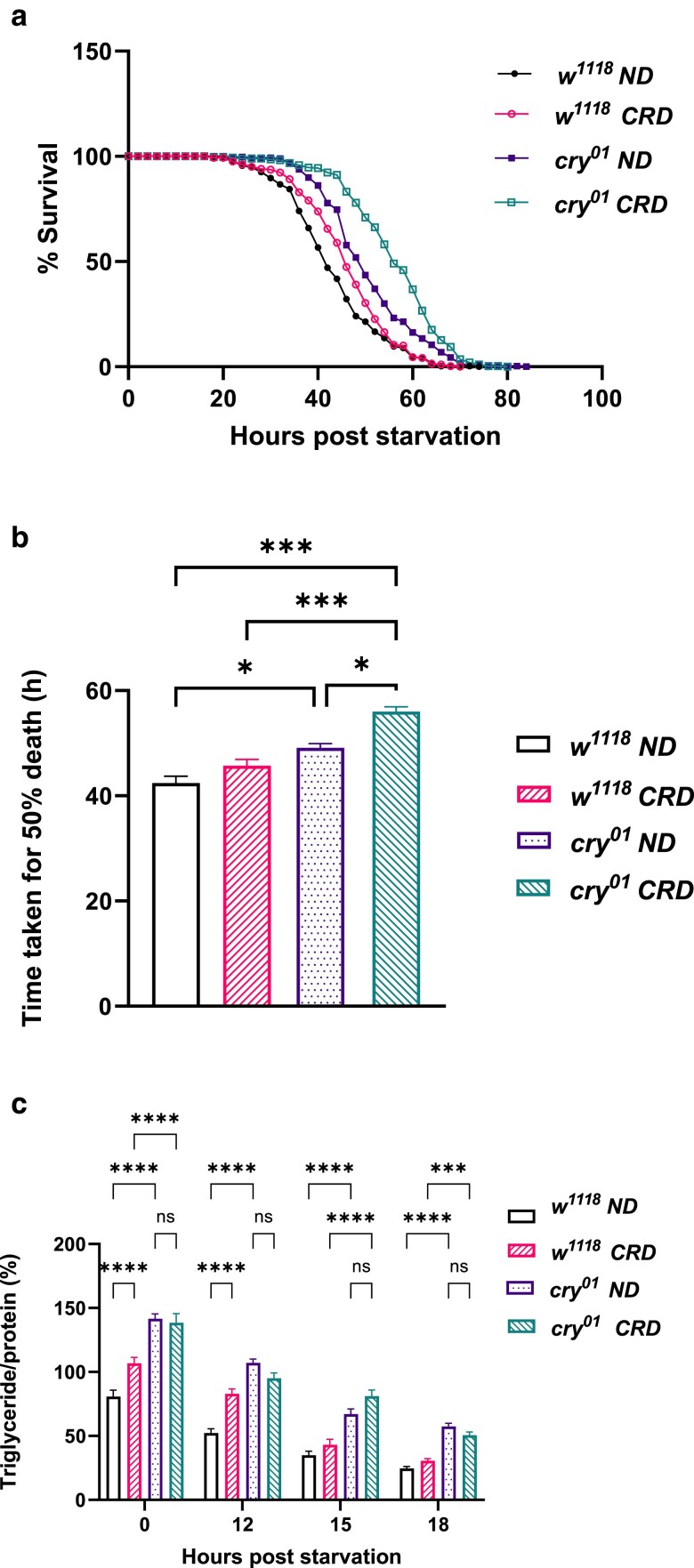
Effect of calorie restriction on starvation sensitivity and TG utilization in *cry* mutant flies. a) Survival curve for *w^1118^* and *cry^01^* flies under LD fed with ND and CRD, data shown as the percentage of flies which were alive at various time points of starvation. b) Time taken for 50% fly death under starvation for *w^1118^* and *cry^01^* flies under LD fed with ND and CRD. c) TG utilization in *w^1118^* and *cry^01^* flies under LD fed with ND and CRD at 00, 12, 15, and 18 h poststarvation.

Since we observed a significant difference in starvation sensitivity and TG levels between *cry^01^* and *w^1118^* flies, we further investigated the effects of dietary modulation on TG metabolism and whether *cry* plays any role in it. We first wanted to see whether *cry* plays any role in regulating metabolism when the flies are fed with a Calorie restricted diet (Rehman and Varghese 2021), henceforth referred to as CRD. This diet was fed to both *cry^01^* and *w^1118^* flies for 5 days postadult emergence. Ad libitum*-*fed (referred to as ND) *cry^01^* and *w^1118^* flies were used as controls. When starvation sensitivity was assayed, we observed that both *cry^01^* and *w^1118^* flies fed with CRD were significantly resistant to starvation stress compared with their ND-fed counterparts (Fig. 5a, *P* = 0.005 for *w^1118^* ND vs *w^1118^* CRD by log-rank test, *P* < 0.0001 for *cry^01^* ND vs *cry^01^* CRD by Log-rank test). When the time taken for 50% fly death was calculated, it was significantly more for *cry^01^* flies fed with CRD compared with *cry^01^* flies fed with ND and there was no significant difference between *w^1118^* flies fed with ND and CRD (Fig. 5b, *P* = 0.03 for *cry^01^* ND vs *cry^01^* CRD). *cry^01^* flies fed with both ND and CRD had increased starvation resistance compared with *w^1118^* flies (Fig. 5a, *P* < 0.0001 by log-rank test). There was a significant difference in the time taken for 50% fly death between *w^1118^* and *cry^01^* flies fed with ND (Fig. 5b, *P* < 0.02 by Kruskal–Wallis test) and *w^1118^* and *cry^01^* flies fed with CRD (Fig. 5b, *P* < 0.001 by Kruskal–Wallis test).

When the TG utilization was measured, *w^1118^* flies fed with CRD had increased TG levels compared with *w^1118^* flies fed with ND at 00 and 12 h poststarvation (Fig. 5c, *P* < 0.001 for *w^1118^* ND vs *w^1118^* CRD at 00 and 12 h, two-way ANOVA). *cry^01^* flies fed with ND had higher TG levels compared with *w^1118^* flies fed with ND at 00, 12, 15, and 18 h poststarvation (Fig. 5c, *P* < 0.0001 for *cry^01^* ND vs *w^1118^* ND at 00, 12, 15 h, and *P* < 0.001 for *cry^01^* ND vs *w^1118^* ND at 18 h, two-way ANOVA). *cry^01^* flies fed with CRD had higher TG levels compared with *w^1118^* flies fed with CRD at 00, 15, and 18 h poststarvation (Fig. 5c, *P* < 0.0001 for *cry^01^* CRD vs *w^1118^* CRD at 00, 15, and 18 h, two-way ANOVA). However, there was no significant difference in the TG levels between *cry^01^* flies fed with ND and CRD (Fig. 5c), indicating a possible role for CRY in governing the TG levels under calorie restriction.

### Role of CRYPTOCHROME in regulating TG levels under a HFD

**Fig. 6.**
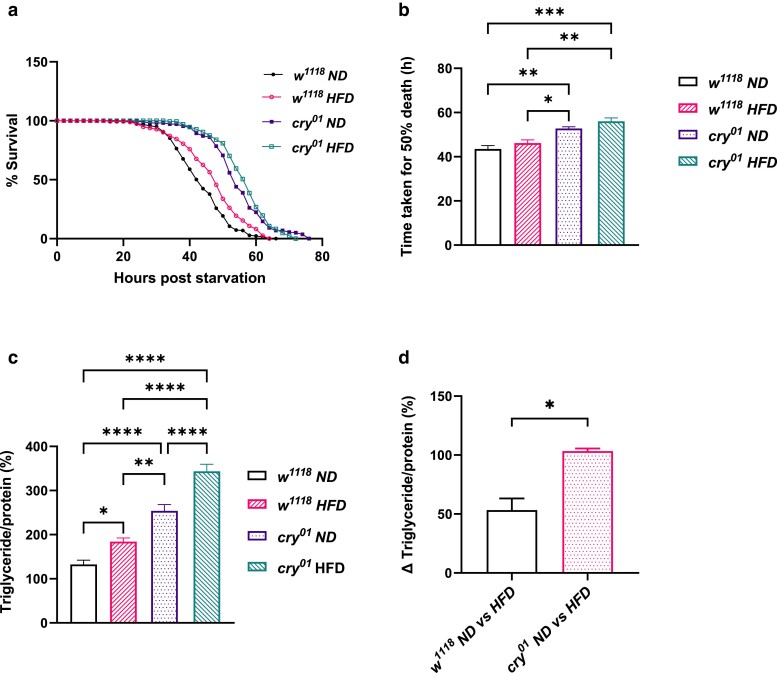
Effect of HFD on starvation sensitivity and TG levels in *cry* mutant flies. a) Survival curve for *w^1118^* and *cry^01^* flies under LD fed with HFD and ND, data shown as the percentage of flies which were alive at various time points of starvation. b) Time taken for 50% fly death under starvation for *w^1118^* and *cry^01^* flies under LD fed with HFD and ND. c) TG levels in *w^1118^* and *cry^01^* flies under LD fed with ND and HFD. d) Δtriglyceride/protein (%) in *w^1118^* ND vs HFD and *cry^01^* ND vs HFD.

We also wanted to see whether *cry* affects metabolism when the flies are fed with a HFD. Sensitivity to starvation and TG levels were assayed in 5-day-old *w^1118^* and *cry^01^* male flies fed with a HFD after adult emergence (refer to *Methods* for the media composition). Controls were ad libitum-fed (referred to as the ND) *w^1118^* and *cry^01^* flies. Both the *w^1118^* and *cry^01^* flies fed with HFD had increased resistance to starvation compared with their respective controls fed with ND (Fig. 6a, *P* < 0.0001 for *w^1118^* ND vs *w^1118^* HFD, *P* < 0.001 for *cry^01^* ND vs *cry^01^* HFD by log-rank test). However, there was no significant difference in the time taken for 50% fly death between *w^1118^* flies fed with HFD and ND and between *cry^01^* flies fed with HFD and ND (Fig. 6b). *cry^01^* flies had higher starvation resistance compared with *w^1118^* flies under both HFD and ND (Fig. 6a, *P* < 0.0001 for *w^1118^* ND vs *cry^01^* ND, *P* < 0.0001 for *w^1118^* HFD vs *cry^01^* HFD by log-rank test). We also saw a significant difference in the time taken for 50% fly death between *cry^01^* flies fed with HFD and ND and *w^1118^* flies fed with HFD and ND, respectively (Fig. 6b, *P* = 0.001 for *w^1118^* ND vs *cry^01^* ND, *P* = 0.001 for *w^1118^* HFD vs *cry^01^* HFD by Kruskal–Wallis test).

On assaying TG levels, we observed that both *w^1118^* and *cry^01^* flies fed with HFD had increased TG levels compared with their ND-fed counterparts (Fig. 6c, *P* = 0.032 for *w^1118^* ND vs *w^1118^* HFD, *P* < 0.0001 for *cry^01^* ND vs *cry^01^* HFD by Kruskal–Wallis test). *cry^01^* flies fed with HFD had increased TG levels compared with *cry^01^* flies fed with ND, and a similar trend was observed with *w^1118^* flies fed with HFD. When the difference in the accumulation of TGs (ΔTG) between the flies fed with ND and HFD was quantified, there was a significant increase in the accumulation of the TGs in *cry^01^* flies compared with *w^1118^* flies (Fig. 6d, *P* = 0.0286 for Δtriglycerides/protein (%) for *w^1118^* ND vs *w^1118^* HFD and *cry^01^* ND vs *cry^01^* HFD by Mann–Whitney test). It seems like *cry* plays a role in modulating the amount of TGs stored when the flies are fed with HFD.

## Discussion

Multiple studies have demonstrated that the circadian clock is indispensable for maintaining metabolic homeostasis (Barber *et al*. 2016; Rhoades *et al*. 2018). Conversely, metabolic signals provide feedback to the circadian timekeeping system to maintain the robustness of the circadian rhythms (Zheng and Sehgal 2010; Katewa *et al.* 2016). The circadian clock rhythmically activates and represses several genes involved in lipid biosynthesis and fatty acid oxidation through clock proteins in mammals (Gooley 2016). Several metabolomic studies have characterized the widespread effects of a disrupted circadian clock on metabolism in *Drosophila*, including its impact on lipids (DiAngelo *et al*. 2011; Rhoades *et al*. 2018; Schäbler *et al*. 2020). This study aimed to probe into a possible unconventional role for the primary circadian photoreceptor CRYPTOCHROME in metabolism.

In *Drosophila*, *cry* transcript levels start out very low in the larval and pupal stages, gradually increase after the adult emergence from day 1 and remain relatively unchanged from day 5 up to day 30 (Graveley *et al.* 2011). Also, since 70% of the larval fat present in the freshly emerged flies is utilized within 4–5 days (Rehman and Varghese 2021), 5-day-old flies were used to test the starvation sensitivity and for all our metabolic assays. The starvation sensitivity of *Drosophila* is a complex trait influenced particularly by energy reserves such as glycogen and lipids—stored in the form of TGs in the fat body, which are critical for survival during starvation (Gibbs and Reynolds 2012). During the initial phase of starvation, within the first 24 h, there is a significant reduction in TG levels as lipolysis is highly active to maintain energy production (Rehman and Varghese 2021). The *cry^01^* flies fared better under starvation, started out with increased TG levels and continued to have higher levels of TGs at 12 and 15 h poststarvation compared with the controls, indicating that the lipid metabolism might be altered in *cry^01^* flies (Fig. 1a-c).

The results of our study hint toward an altered carbohydrate metabolism in *cry^01^* flies in addition to an altered TG metabolism (Fig. 1g). Although we observed increased TG and glycogen levels in *cryptochrome* mutants, the results previously reported by Seay and Thummel (2011) showed that *cry^01^* flies had reduced triacylglyceride and glycogen levels compared with the wild-type flies. We speculate that it could be owing to the differences in the control strain (*Berlin-K*) and the media composition used for rearing the flies for 5 days under LD. Yeast paste made with 1% dextrose solution was used in Seay and Thummel (2011) and standard cornmeal dextrose medium was used in our study. In mammals, CRY has been shown to regulate hepatic glucose production (Zhang *et al*. 2010). Studies have also indicated that CRY inhibits glucose metabolism by repressing transcription of metabolic genes, including the glucocorticoid receptor (Lamia *et al*. 2011). These insights, alongside our study, underscore the complex interplay between the CRY and carbohydrate metabolism.

Feeding in flies is rhythmic and the timing is controlled by the circadian clocks (Xu *et al.* 2008). *cry^01^* flies seemed to feed more at ZT01 compared with the control flies under LD which could possibly be one of the reasons for the increase in resistance to starvation (Lee and Jang 2014) and TG/protein levels that were seen (Fig. 1h). Under LL, clock-mediated rhythmic processes are affected since the external time cues are absent (Marrus *et al*. 1996) and this led to the rhythmic feeding being abolished in *w^1118^* flies. *cry^01^* flies show increased food consumption compared with the control under LL. It remains to be seen whether *cry^01^* flies exhibit any rhythms in feeding under LL akin to the split activity-rest rhythms they display under LL.

The circadian feeding rhythms in *Drosophila* are also regulated by the neuronal populations in the PI region of the brain expressing SIFamide and DILPs, highlighting how the circadian clock and the insulin signaling cascade are intricately linked (Dreyer *et al.* 2019). Insulin signaling influences lipid and carbohydrate metabolism as well as the size of flies (Gillette *et al.* 2021). The insulin-regulated transcription factor FOXO is a crucial player in the insulin signaling cascade, and in *cry^01^* flies, we observed reduced transcript levels of the FOXO targets *4ebp* and *dilp6* (Fig. 2b, c). DILP2 exerts its effect on metabolism mainly by signaling through the *Drosophila* insulin receptors (InRs) expressed in the fat body. Thus, insulin signaling regulates the lipid storage in the fat body (Chatterjee and Perrimon 2021). The fat body plays a major role in this signaling by relaying important information about the nutritional status (Texada *et al.* 2019) through molecules such as DILP6. It is possible that the increase in stored energy reserves we see in *cry^01^* flies is due to augmented insulin signaling (Britton *et al.* 2002; DiAngelo and Birnbaum 2009). We would further need to probe into the circulating glucose, trehalose, and DILP2 levels in the hemolymph to verify the role of *cry* in insulin signaling. Response to starvation stress in *Drosophila* is also influenced by genetic, developmental, and environmental factors (Rion and Kawecki 2007). Therefore, the observed starvation resistance in *cry^01^* flies is likely also attributable to other metabolic processes and environmental factors, in addition to insulin signaling.

The metabolic changes we observed in *cry^01^* flies likely depend on an intact circadian clock as evidenced by the reversal of these effects such as increased starvation sensitivity and reduced TG levels in *per^01^;;cry^01^* double mutants when fed ad libitum (Fig. 3a-c). Although the TG levels in the *per^01^;;cry^01^* double mutants are not significantly different than that of *per^01^* flies when fed ad libitum, they seem to show a difference in TG utilization during the course of starvation which could be due to the differences in how the energy reserves such as TGs and glycogen are utilized under starvation in these flies. Previously it has been shown that the clock gene *per* in *Drosophila* affects intermediary lipid metabolism and renders the flies susceptible to starvation (Schäbler *et al*. 2020). *per^01^* clock mutants have also been shown to have impaired metabolite cycling (Amatobi *et al*. 2023). This could partially explain the reduced starvation resistance and TG levels we observed in the *per^01^;;cry^01^* double mutants. However, the double mutants showed increased starvation sensitivity compared with the control rather than rescuing the starvation resistance phenotype observed in *cry^01^* flies. The levels of *tim* transcript may vary between *per^01^*, *cry^01^*, and *per^01^;;cry^01^* flies. Therefore, it is crucial to understand the role of *tim* in CRY-mediated TG metabolism. CRY has also been found to act as a transcriptional repressor in the eye clock when overexpressed. It was seen that both CRY and PER independently exert their effects on different steps that repress CLK/CYC activity (Collins *et al.* 2006).

It is also interesting to note that while *per^01^* mutants suppress the *cry^01^* phenotype, LL does not suppress the *cry^01^* metabolic phenotype, although LL disrupts the circadian clock and degrades CRY. We expected that the differences in the metabolic phenotypes we observed under LD between *w^1118^* and *cry^01^* flies might be attenuated under LL, since CRY is degraded in the presence of light and the control flies would have reduced levels of CRY under LL compared with LD. However, we still saw a significant difference in the starvation resistance and TG utilization between the *w^1118^* and *cry^01^* flies reared under LL (Fig. 1d-f). It is important to understand whether the role of CRY in TG metabolism is independent of its function in the light entrainment of the activity-rest rhythm. When flies are kept under LL, they typically exhibit arrhythmicity in activity-rest rhythm since the CRY is degraded and PER-TIM cycling is affected (Collins and Blau 2007). Conversely, flies defective for *cry* exhibit split behavioral rhythm under LL (Dolezelova *et al.* 2007). In *Drosophila*, morning and evening peak activities derive from 2 distinct groups of coupled circadian oscillators (Grima *et al*. 2004; Stoleru *et al*. 2004). Flies overexpressing the pacemaker gene *per* in a subset of DN1 s were rhythmic under LL indicating these neurons’ importance in modulating the behavioral rhythm in response to LL (Murad *et al*. 2007). While CRY and PER expressed in the circadian pacemaker govern the activity-rest rhythm under LL, the specific roles of CRY and PER in the circadian pacemaker and peripheral clocks in governing the lipid metabolism under LD and LL remain to be understood. The overarching effect of LL on metabolic processes and the clock-independent mechanisms that affect metabolism and energy homeostasis in flies under LL also need to be taken into account (Yang *et al.* 2022).

While addressing the central and peripheral clock specific roles of CRY and PER in metabolism, it is important to note a previous study where downregulation of a core circadian clock gene *Clk* in the central pacemaker neurons appears to increase the fat body TG levels (DiAngelo *et al*. 2011). Xu *et al.* (2008) demonstrated the existence of a functional clock in the *Drosophila* fat body; the seat of stored energy reserves such as TGs and glycogen and is vital for metabolic homeostasis in *Drosophila*. A follow-up study in 2011 (Xu *et al.* 2011) reported that *cry* was one of the many cyclically expressed genes in the fat body. CRY is also expressed more abundantly in the gut than in the fat body (Leader *et al.* 2018). A systemic study on fly internal organs showed that *per* and *tim* cycle in peripheral tissues, including the alimentary tract and fat body (Zhao *et al*. 2019; Giebultowicz *et al*. 2001). Further investigation is needed to understand the role of CRY and PER expressed in peripheral tissues on the metabolic phenotypes we observe in *cry^01^* and *per^01^;;cry^01^* flies. We would also need to assess how the peripheral clock oscillations in the gut and fat body are affected in these flies. Also, it remains to be seen whether the effects we observed in *cry^01^* and *per^01^;;cry^01^* flies are because of the role of CRY in the central or the peripheral clocks or in tandem effects.

Among the various zeitgebers that entrain the circadian clock, food is considered as a weaker zeitgeber for the central clock. Previous studies have examined whether food can entrain the circadian rhythm in *Drosophila*, revealing that restricted feeding indeed drives the rhythmic expression of clock genes in the fat body, but not in the central clock (Xu *et al*. 2011). Food is also considered as an entraining stimulus for metabolic rhythms (Mistlberger 2011), and the food-entrainable oscillators may operate through mechanisms distinct from those of light-entrainable oscillators (Mistlberger 2011). Therefore, it is important to distinguish the extent to which the different CRY-expressing peripheral clocks in metabolic tissues rely on light and food for regulating the TG metabolism. Previous studies indicate photoreceptor-independent roles of CRY in specific tissues which are regulated by different molecular mechanisms (Damulewicz and Mazzotta 2020). Examining the tissue-specific role of CRY in governing metabolism will help us understand this better.

Aging and lifespan in flies are influenced by multiple factors such as diet and energy utilization to name a few and a number of underlying molecular players (Piper and Partridge 2018). In addition, previous studies have shown a strong link between the circadian clock and longevity (Klarsfeld and Rouyer 1998; Solovev *et al*. 2019). The role of the circadian clock in regulating energy metabolism further underscores its impact on aging and lifespan (Froy 2013). Mounting evidence also indicates that dietary interventions such as different feeding regimens robustly affect the circadian clock machinery and subsequent clock-controlled metabolic pathways and longevity (Froy 2011, 2013, 2018). We did observe a remarkable increase in lifespan in *cry^01^* flies compared with *w^1118^* flies (Fig. 4a, b). The tradeoffs for the increased lifespan we observe in the *cry^01^* flies also need to be studied. Previously, a study by Ozturk *et al.* (2009) showed that *Cry* mutation in mice reduces cancer risk and extends their median lifespan. Although we have not probed into the possible underlying mechanisms for such an increase in the observed lifespan in a *cry* mutant background in *Drosophila*, we speculate that it could be partly due to the increased levels of energy reserves (TGs and glycogen). Studies have shown that continuous light exposure can disrupt circadian rhythms, increase oxidative stress, and impair various physiological processes, ultimately leading to reduced lifespan in *Drosophila* (Sheeba *et al.* 2000; Nash *et al.* 2019; Song *et al.* 2022). Hence, differences in light-sensing abilities between the *w^1118^* and *cry^01^* flies could also contribute to the longevity enhancement observed.

Calorie restriction is a well-studied dietary modulation technique and has been shown to extend lifespan in flies by conferring better health span, stress resistance, and delaying the onset of aging (Li *et al.* 2023; Partridge *et al.* 2005). A study also pointed out the role played by the circadian clock in the extension of lifespan under caloric restriction in flies (Hodge *et al*. 2022; Hwangbo *et al.* 2023). Moreover, restricting food availability to a 6-h interval each day drives rhythmic expression of genes related to metabolism, detoxification, the immune response, and steroid hormone regulation in the fat body (Xu *et al.* 2011). When the *cry^01^* flies were fed with a CRD, we observed that the flies had increased resistance to starvation (Fig. 5a, b). It has been shown previously that under caloric restriction, flies increase their fatty acid synthesis and breakdown which in turn alters the steady-state whole-body TG levels and the TG turnover rates (Katewa *et al*. 2012). In accordance with this, we observed that *w^1118^* flies fed with a CRD had more TG levels compared with *w^1118^* flies fed with a ND (Fig. 5c). We speculate that the *cry* mutation has had an effect on how flies respond to a deficit in calories owing to which we do not see a difference in the TG levels. Further studies on glycogen levels are required to better understand the possible reasons we see an increased starvation resistance in *cry^01^* flies fed with a CRD. Understanding how calorie restriction and circadian clock interact to regulate TG and glycogen levels may give insights into the importance of the circadian timing system for organisms in adapting to changes in nutrient availability and daily rhythms.

Feeding flies with a HFD for prolonged periods has been proven to be detrimental in a lot of ways affecting behavior, metabolism, fecundity, and lifespan (Liao *et al.* 2021). A recent study showed altered expression of levels of core clock genes *per*, *tim*, and *clock* in *Drosophila* under HFD (Nayak and Mishra 2021). Both *w^1118^* and *cry^01^* flies fed with HFD showed enhanced TG levels compared with the *w^1118^* and *cry^01^* flies fed with ND although they do not exhibit a marked difference in starvation resistance (Fig. 6b, c). It is possible that *cry^01^* flies are not effectively utilizing the excess TGs during the course of starvation since the *cry^01^* flies seemed to have more TG accumulation under HFD compared with the *w^1118^* flies (Fig. 6d). Previous studies on *Clock* mutant mice showed less TG accumulation in the liver and impaired dietary fat absorption under high-fat diet (HFD) (Oishi *et al.* 2006; Kudo *et al.* 2007). Furthermore, ablation of *Cry1*, prevented HFD induced obesity in mice. Although serum lipid and glucose profiles showed no difference between *Cry1^−/−^* and wild-type mice (Griebel *et al.* 2014), the results of our study suggest a role for CRY in regulating TG storage in *Drosophila* under HFD. These studies reinforce the important role of circadian clock genes in energy homeostasis under HFD. Further, it is important to understand the underlying pathways by which *cry* regulates energy storage in response to dietary changes.

From the results of this study, we speculate that *Drosophila* CRYPTOCHROME could be moonlighting as a regulator of metabolism in peripheral tissues. The present study did not look into the tissue-specific roles of CRY expressed in the central and peripheral clocks in governing TG metabolism. Further studies are necessary to better understand the importance of CRY-expressing peripheral clocks in the metabolic tissues.

## Supplementary Material

jkae220_Supplementary_Data

## Data Availability

Supplementary Table 1 lists the primers used in the study. File S1 contains all the relevant raw data. The fly lines used were obtained from BDSC (*w^1118^*), Dr Sheeba Vasu, JNCASR (*cry^01^*), and Dr Charlotte Förster, University of Wurzburg [*per^01^*, *cry^01^ (cantonized)*, and *per^01^;;cry^01^*]. Supplemental material available at G3 online.
