# Supplementary material for: A role for the circadian photoreceptor CRYPTOCHROME in regulating triglyceride metabolism in Drosophila
Source: G3 (Bethesda). 2024 Sep 12;14(11):jkae220. doi: 10.1093/g3journal/jkae220 (PMC11540332; doi:10.1093/g3journal/jkae220)
Supplement: jkae220_Supplementary_Data [file jkae220_supplementary_data.zip › Supplemental_Material_Legends_G3-2024-405355.docx]

**Figure S1.** (A) Relative mRNA abundance of *cry* in *w^1118^* and *cry^01^* heads at ZT02, values in parentheses indicate mean ± SEM. (B, C) Representative actograms of *w^1118^* and *cry^01^* under LD-LL. (D) Weights of the flies (in g) - *w^1118^* and *cry*^01^*.* (E) Relative mRNA abundance of *cry* in *Canton-S, cry^01^ and per^01^;;cry^01^* heads at ZT02, values in parentheses indicate mean ± SEM (F) Agarose gel electrophoresis image of PCR amplified DNA; M-1Kb DNA ladder, 1-*Canton-S*, 2-*per^01^;;cry^01^*, 3-*per^01^* (G) Agarose gel electrophoresis image of PCR product after restriction digestion with XbaI; DNA; M-100bp DNA ladder, 1-*Canton-S*, 2-*per^01^*, 3-*per^01^;;cry^01^*

**Table S1** List of primers used.

**Table S2** Statistical analysis details for Figure 3C.
