# Supplementary figures and images for: A role for the circadian photoreceptor CRYPTOCHROME in regulating triglyceride metabolism in Drosophila
Source: G3 (Bethesda). 2024 Sep 12;14(11):jkae220. doi: 10.1093/g3journal/jkae220 (PMC11540332; doi:10.1093/g3journal/jkae220)

**Figure S1**

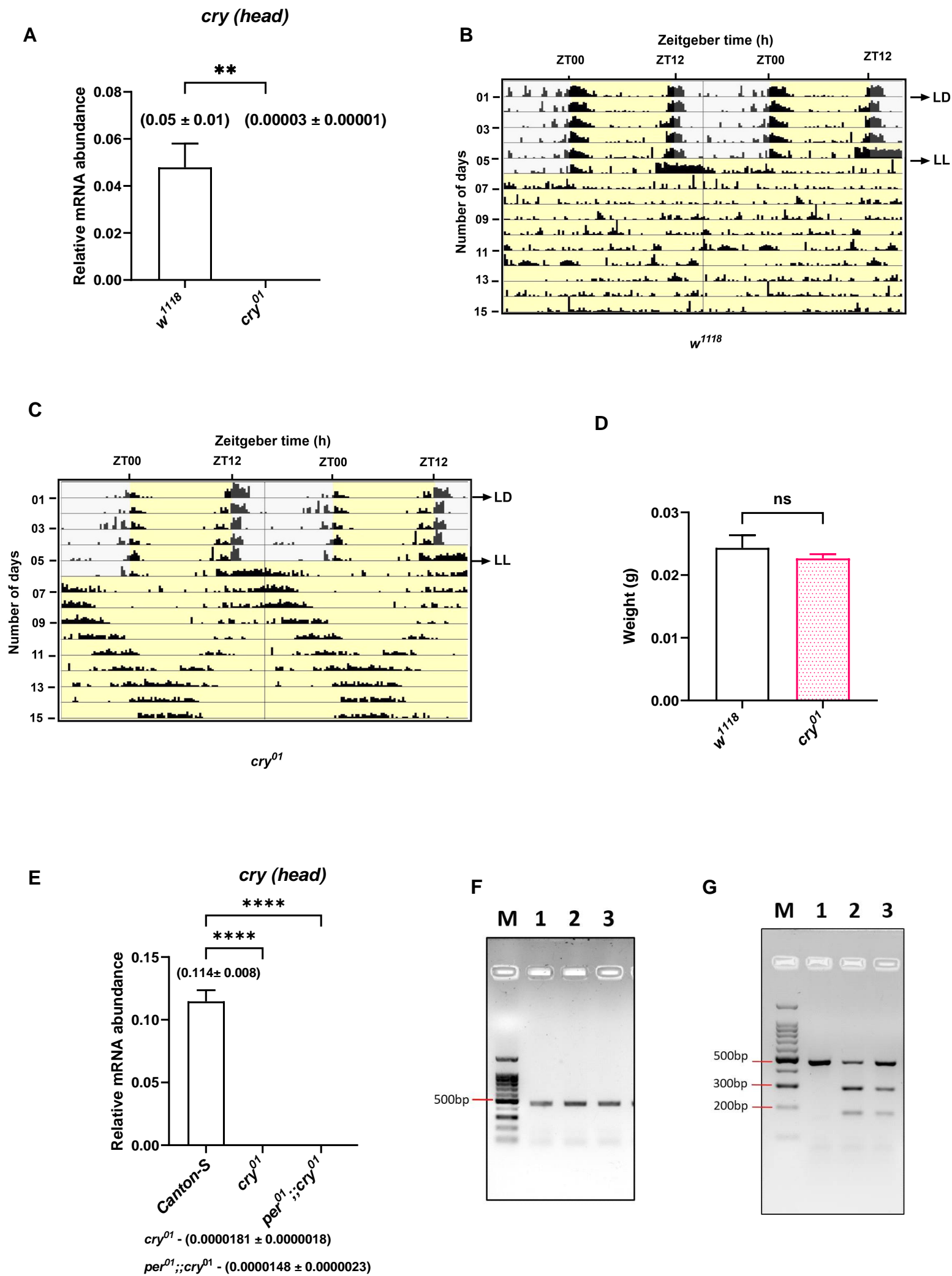

Supplement: jkae220_Supplementary_Data [file jkae220_supplementary_data.zip › Figure_S1_G3-2024-405355.pdf]
